# HOTTIP-miR-205-ZEB2 Axis Confers Cisplatin Resistance to Ovarian Cancer Cells

**DOI:** 10.3389/fcell.2021.707424

**Published:** 2021-07-12

**Authors:** Yu-Jie Dong, Wei Feng, Yan Li

**Affiliations:** ^1^Department of Emergency, China-Japan Union Hospital of Jilin University, Changchun, China; ^2^Department of Anesthesiology, China-Japan Union Hospital of Jilin University, Changchun, China

**Keywords:** ovarian cancer, cisplatin resistance, HOTTIP, miR-205, ZEB2

## Abstract

Ovarian cancer is a deadly gynecological malignancy with resistance to cisplatin a major clinical problem. We evaluated a role of long non-coding (lnc) RNA HOTTIP (HOXA transcript at the distal tip) in the cisplatin resistance of ovarian cancer cells, using paired cisplatin sensitive and resistant A2780 cells along with the SK-OV-3 cells. HOTTIP was significantly elevated in cisplatin resistant cells and its silencing reversed the cisplatin resistance of resistant cells. HOTTIP was found to sponge miR-205 and therefore HOTTIP silenced cells had higher levels of miR-205. Downregulation of miR-205 could attenuate HOTTIP-silencing effects whereas miR-205 upregulation in resistant cells was found to re-sensitize cells to cisplatin. HOTTIP silencing also led to reduced NF-κB activation, clonogenic potential and the reduced expression of stem cell markers SOX2, OCT4, and NANOG, an effect that could be attenuated by miR-205. Finally, ZEB2 was identified as the gene target of miR-205, thus completing the elucidation of HOTTIP-miR-205-ZEB2 as the novel axis which is functionally involved in the determination of cisplatin resistance in ovarian cancer cells.

## Introduction

Ovarian cancers are a group of heterogenous malignancies and are one of the deadliest gynecological cancers with five-year survival close to or less than 50% (Torre et al., 2018). In Chinese population, mortality due to ovarian cancer is rapidly increasing (He et al., 2021). The heterogenous nature of ovarian cancer calls for novel studies to fully understand the disease etiology and identify novel targets of therapy. Clinical management of ovarian cancer involves the use of cisplatin (Bergamini et al., 2017) either alone or in combination with other drugs (Lee et al., 2020). However, resistance to cisplatin is a common clinical observation in patients being treated with cisplatin (Yang et al., 2020). In the fight against ovarian cancer and in view of the importance of cisplatin in the treatment of ovarian cancer patients, a better understanding of resistance mechanisms will undoubtedly be important.

In addition to the exploration of various genetic and pathway-based mechanisms for resistance to cisplatin, efforts are underway to understand the epigenetic mechanisms of cisplatin resistance with long non-coding (lnc) RNAs as the molecules of interest (Vera et al., 2018). The differential expression of lncRNAs can help maintain the balance between cisplatin resistance and sensitivity (Li et al., 2018; Taheri et al., 2021). The lncRNAs-based epigenetic signature can also possibly help stratify ovarian cancer patients with implications in precision medicine (Liu et al., 2017). A number of lncRNAs, such as HOTAIR (Wang Y. et al., 2015; Li et al., 2016; Yu et al., 2018; Zhang et al., 2020) and MALAT1 (Bai et al., 2018; Wang Y. et al., 2020; Taheri et al., 2021) have been investigated for their possible role in regulating sensitivity to cisplatin of ovarian cancers. In this study, we hypothesized a possible role of lncRNA HOTTIP (HOXA transcript at the distal tip) in the cisplatin resistance of ovarian cancer cells. HOTTIP has been implicated in cisplatin resistance of pancreatic cancer cells (Yin et al., 2020) which would suggest its similar role in other cancers, such as ovarian cancer as well, but to-date there has been no report on the subject. In our study, we used a paired cell line comprising of parental A2780 ovarian cancer cells and the cisplatin resistant A2780 cells (A2780-CR). The parental cells are sensitive to cisplatin while the derivative cisplatin resistance cells are resistant. In addition, we used SK-OV-3 for further validation of our findings. These cells are relatively resistant to cisplatin, as compared to the A2780 cells. Moreover, we focused on understanding the mechanism of HOTTIP-mediated generation of cisplatin resistance by identifying the miRNA that it sponges as well as the downstream gene target. Based on our findings, we believe that HOTTIP-miR-205-ZEB2 axis plays a critical role in cisplatin resistance of ovarian cancer cells.

## Materials and Methods

### Cell Lines

Ovarian cancer cells A2780 cells and their cisplatin-resistant derivatives (referred in this study as A2780-CR) were obtained from Sigma (St Louis, MO, United States) while SK-OV-3 cells were from ATCC (Manassas, VA, United States). Cells were cultured in RPMI 1640 media with 10% fetal bovine serum and 1% antibiotics in a 5% CO_2_-humidified atmosphere at 37°C. The si-HOTTIP as well as si-ZEB2 was purchased from Shanghai GenePharma Co., Ltd. (China).

### RT-PCR for lncRNA, miR, and mRNA Detection

Total RNA was isolated using the mirVana miRNA isolation kit (Ambion, United States) as per instructions. The quality of RNA was checked and the RNA quantitated using NanoDrop instrument. Ten nanogram samples were used for the quantitation of miR-205. For mRNA detection, 1 μg RNA was used to prepare cDNA before detection of individual genes using SYBR Green based detections and using GAPDH as the internal control. lncRNA HOTTIP and miR-205 levels were determined using reagents from Thermo Fisher Scientific (United States). mRNA PCR was run on StepOne Applied Biosystems real-time PCR instrument.

### Pre/anti-miR Transfections

Pre- and anti-miR-205 oligos were purchased from Thermo Fisher Scientific (United States) and transfected in cells at 20 nM concentrations using Lipofectamine 3000 (Invitrogen, China). Transfected cells were allowed to grow for 72 h and then subjected to another round of transfections. Cells were transfected at least three times before being used in the experiments.

### Cell Proliferation Assay

Cell Proliferation Assay kit was from ATCC (Manassas, VA, United States). Tetrazolium MTT (3-(4, 5-dimethylthiazolyl-2)-2, 5-diphenyltetrazolium bromide) was reduced by cells that are metabolically active, due to the action of dehydrogenase enzymes, resulting in generation of reducing equivalents NADH and NADPH. Cells were seeded overnight in 96 well plates and then treated as explained for each experiment. Then, 10 μl MTT reagent was added for 2 h, followed by the supplied detergent reagent (100 μl) for 4 h. Plates were read at 575 nm in a plate reader (Shimadzu, Japan).

### Clonogenic Assay

For the anchorage-dependent clonogenic assay, ovarian cancer cells A2780 and SK-OV-3 cells were counted and resuspended in complete culture medium to obtain single cell suspensions. Cells were seeded overnight in six-well plates at a density of 750 cells per well. After 3 weeks of growth in an incubator under 5% O_2_, 5% CO_2_, and 90% N_2_ conditions, colonies were fixed with 4% paraformaldehyde, stained with crystal violet and counted.

For the anchorage-independent clonogenic assay, ovarian cancer cells A2780 and SK-OV-3 cells were counted and resuspended in complete culture medium to obtain single cell suspensions. Cells were then suspended in cell media containing 0.7% top agar which was layered over a base layer consisting of 0.8% base agar. Cells were cultured in a 5% CO_2_-humidified atmosphere at 37°C for 4–5 weeks at the end of which the colonies were manually counted under a microscope.

### NF-κB p65 Activation Assay

The kit was purchased from Abcam. This kit semi-quantitatively assays NF-κB. The principle of this assay is that the 96-well plate comes with immobilized double stranded DNA sequence containing the NF-κB response element. When nuclear extracts with activated NF-κB are added to the plate, NF-κB binds to the NF-κB response element and is detected using a specific antibody against NF-κB. Thereafter, a secondary HRP-conjugated antibody is added to enable colorimetric readout at 450 nm. In our assays, subsequent to the individual experimental conditions, nuclear extracts were prepared and equal amount of samples were added to different wells of the purchased 96-well plate. The plate was left overnight and then washings and additions of primary and secondary antibodies were done, as per manufacturer’s instructions. Incubations with primary as well as secondary antibodies were for 1 h each at room temperature. Microplate reader (Shimadzu, Japan) was used to record absorbance at 450 nm.

### Statistical Analysis

All reported results are representative of at least three independent experiments. We used student’s *t*-test to evaluate the level of significant differences between group means, and performed statistical analysis using Prism 5 (GraphPad software). A *p* value of <0.05 was considered significant.

## Results

### HOTTIP Is Elevated in Cisplatin Resistant Cells

**FIGURE 1:**
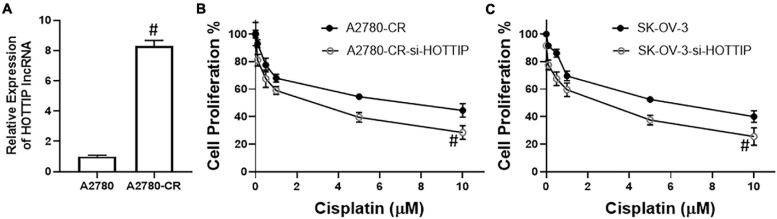
**(A)** LncRNA HOTTIP was measured in parental A2780 and derivative cisplatin resistant A2780-CR cells by RT-PCR. Cell proliferation of **(B)** A2780-CR and **(C)** SK-OV-3 cells was measured by MTT assay, as described in section “Materials and Methods.” Cells were treated with indicated final doses of cisplatin for 72 h before the MTT assay. ^#^*p* < 0.05.

To check if our hypothesis for the possible role of HOTTIP in cisplatin resistance of ovarian cancer cells was correct, we measured the levels of HOTTIP in a paired cell line model that comprised of cisplatin sensitive A2780 cells and their cisplatin resistance derivatives (A2780-CR). We found that HOTTIP was expressed at significantly high levels in the resistant cells (Figure 1A) thus confirming our basic hypothesis. Next, we wanted to check if the elevated levels of HOTTIP were functionally important for cisplatin resistance. To check this, we silenced HOTTIP using a specific siRNA (si-HOTTIP) in the resistance cells and treated them with increasing amounts of cisplatin for 3 days. At the end of treatment, cell proliferation was measured. We found that silencing of HOTTIP significantly decreased the proliferation of A2780-CR cells (Figure 1B). We further tested our results in another cell line—SK-OV-3. These ovarian cancer cells are relatively resistant to cisplatin (compared to A2780 cells) and exhibit higher IC-50 values (6.8 μM for SK-OV-3, as compared to 2.3 μM for A2780 cells). We silenced HOTTIP in these cells as well and studied the effect on cisplatin sensitivity. As seen in Figure 1C, we observed that silencing of HOTTIP sensitized the SK-OV-3 cells to cisplatin.

### HOTTIP Silencing Affects Stem Cells and NF-κB

**FIGURE 2:**
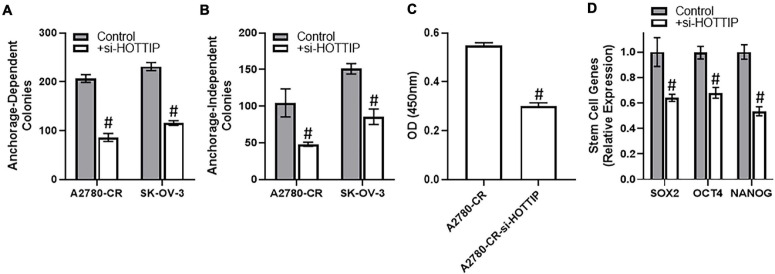
**(A)** Anchorage-dependent and **(B)** anchorage-independent growth of A2780-CR and SK-OV-3 cells with and without silencing of HOTTIP was measured, as mentioned in section “Materials and Methods.” Anchorage dependent growth was allowed for three weeks while anchorage-independent growth in soft agar was allowed for 4–5 weeks. **(C)** NF-κB activity assay was performed using the methods mentioned and the absorbance was read at 450 nm. **(D)** Expression levels of stem cell markers were determined by RT-PCR in A2780 cells with and without the silencing of HOTTIP, with GAPDH being evaluated as internal control. ^#^*p* < 0.05.

To understand the mechanism of HOTTIP action, we turned to cancer stem cell characteristics because of the reports that HOTTIP affects cancer stem cells (Fu et al., 2017; Luo et al., 2019). We checked the clonogenic potential of cells when HOTTIP is silenced. We found that silencing of HOTTIP resulted in significant decrease in the clonogenic potential of both of the cell lines tested, A2780-CR and SK-OV-3. Moreover, the effect was evident on anchorage dependent (Figure 2A) as well as anchorage independent growth (Figure 2B). Since a role of NF-κB is important for cancer stem cells, we evaluated the effect of HOTTIP silencing on NF-κB activation and found that silencing of HOTTIP resulted in significantly reduced activation of NF-κB (Figure 2C). As a direct readout for the effects on stem cell, we checked the characterized markers of stem cells—SOX2, OCT4, and NANOG. It was found that silencing of HOTTIP significantly reduced the levels of SOX2, OCT4, and NANOG genes (Figure 2D).

### HOTTIP Sponges miR-205

**FIGURE 3:**
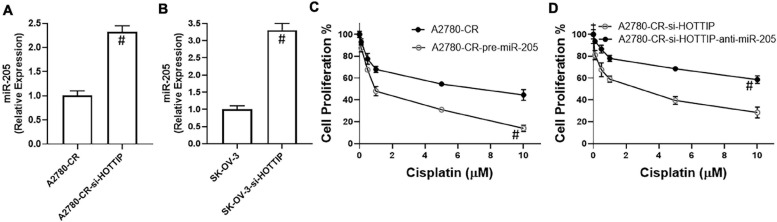
Expression of miR-205 in **(A)** Cisplatin resistant A2780-CR cells (with and without HOTTIP silencing) and **(B)** SK-OV-3 cells (with and without HOTTIP silencing) was quantitated using RT-PCR. **(C)** Cell proliferation for cisplatin resistant A2780-CR with and without pre-miR-205 and **(D)** cell proliferation for HOTTIP silenced A2780-CR cells with and without anti-R-205, was measured by MTT assay. ^#^*p* < 0.05.

The ability of lncRNAs to influence cellular and physiological functions often involves sponging of miRNAs. We found that HOTTIP sponges miR-205 as silencing of HOTTIP significantly increased the levels of miR-205 in A2780-CR cells (Figure 3A). The results were further confirmed in SK-OV-3 cells (Figure 3B). To establish that the observed reciprocal relationship between HOTTIP and miR-205 was because of the sponging of miR-205 by HOTTIP as well as to confirm the role of this relationship in cisplatin resistance, we once again turned to proliferation assay. In the A2780-CR cells, overexpression of miR-205 led to resensitization of these cells to cisplatin (Figure 3C) thus confirming the reciprocal relationship between HOTTIP and miR-205. Since resistant cells, when silenced for HOTTIP, had higher levels of miR-205, we downregulated miR-205 in these cells by using anti-miR-205 oligos in order to further confirm our findings. This resulted in attenuation of HOTTIP silencing effects and cells were once again found to be resistant to cisplatin (Figure 3D).

### miR-205 Effects on Stem Cells and NF-κB

**FIGURE 4:**
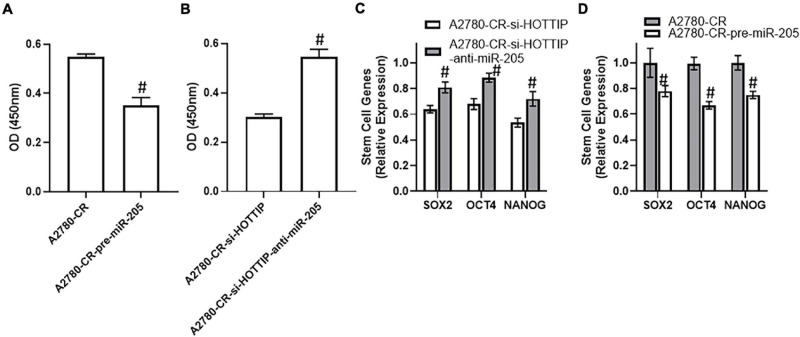
**(A)** NF-kB assay for cisplatin resistant A2780-CR with and without pre-miR-205 and **(B)** NF-kB assay for HOTTIP silenced A2780-CR cells with and without anti-R-205, was performed using commercial kit. **(C)** Expression levels of stem cell makers (SOX2, OCT4 and NANOG) in HOTTIP silenced A2780-CR cells with and without anti-miR-205 and in **(D)** cisplatin resistant A2780-CR with and without pre-miR-205, as determined using a PCR. ^#^*p* < 0.05.

In view of the above findings implicating a role of HOTTIP in stem cell characteristics, we checked its sponging of miR-205 as the underlying cause. Overexpression of miR-205 resulted in significantly reduced NF-κB activation in A2780-CR cells (Figure 4A) and downregulation of miR-205 in HOTTIP silenced resistant A2780 cells had an opposite effect with much more increased activation of NF-κB (Figure 4B). Further, subsequent to downregulation of miR-205 in HOTTIP silenced A2780-CR cells, the levels of stem cell markers SOX2, OCT4, and NANOG significantly increased (Figure 4C) whereas overexpression of miR-205 in A2780-CR cells resulted in decreased expression of SOX2, OCT4, and NANOG (Figure 4D).

### miR-205 Targets ZEB2

**FIGURE 5:**
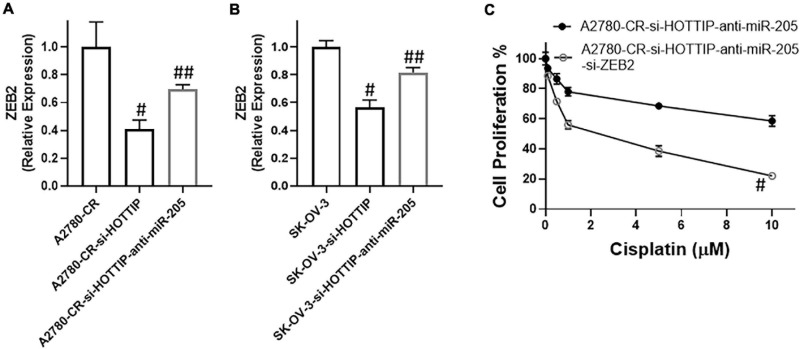
Expression of ZEB2, in **(A)** cisplatin resistant A2780 and **(B)** SK-OV-3 cells under specified conditions (HOTTIP silencing with without anti-miR-205) was measured, using RT-PCR. **(C)** Cell proliferation under specified conditions (HOTTIP silenced A2780-CR cells with anti-miR-205 and additionally with and without si-ZEB2), was measured by MTT assay. ^#^*p* < 0.05 compared to respective controls (A2780-CR/SK-OV-3) ad ^##^*p* < 0.05 compared to HOTTIP-silenced respective cells.

miRNAs function through targeting of their target genes and we found ZEB2 to be target of miR-205 in our study. When miR-205 was downregulated, the levels of ZEB2 went up in the HOTTIP silenced cisplatin resistant A2780-CR cells (Figure 5A). On similar lines and as further confirmation, similar observations were made in SK-OV-3 cells. In these cells as well, downregulation of miR-205 in HOTTIP-silenced cells led to increased expression of ZEB2 (Figure 5B). Furthermore, in both A2780-CR as well as the SK-OV-3 cells, just the silencing of HOTTIP resulted in decreased expression of ZEB2 (Figures 5A,B), even without any manipulations of miR-205 levels, suggesting an influence of HOTTIP on ZEB2 thus establishing a HOTTIP-miR-205-ZEB2 axis. To further confirm the role of this axis in cisplatin sensitivity, we performed proliferation assay and found that silencing of ZEB2 could reverse the effects of miR-205 downregulation (Figure 5C).

## Discussion

Cisplatin resistance remains a major clinical problem. Seeking a possible role of lncRNAs in cisplatin resistance, a number of lncRNAs have already been evaluated for their possible involvement in cisplatin sensitivity/resistance. Examples are UCA1 (Wang F. et al., 2015; Li Z. et al., 2019), HOTAIR (Wang Y. et al., 2015; Li et al., 2016; Yu et al., 2018; Zhang et al., 2020), PVT1 (Liu et al., 2015; Chen et al., 2021), H19 (Zheng et al., 2016; Sajadpoor et al., 2018; Wu et al., 2019), ENST00000457645 (Yan et al., 2017), MEG3 (Zhang J. et al., 2017), ANRIL (Zhang D. et al., 2017; Miao et al., 2019), RP11-135L22.1 (Zou et al., 2018), MALAT1 (Bai et al., 2018; Wang Y. et al., 2020; Taheri et al., 2021), Linc00312 (Zhang et al., 2018), EBIC (Xu et al., 2018), HOXD-AS1 (Chi et al., 2018), PANDAR (Wang et al., 2018), CASC11 (Shen et al., 2019), LINC00152 (Zou and Li, 2019), NCK1-AS1 (Chang et al., 2020), LINC01125 (Guo and Pan, 2019), CCAT1 (Wang D.Y. et al., 2020), NEAT1 (Zhu et al., 2020), CHRF (Tan et al., 2020), ZEB1-AS1 (Dai et al., 2021), TRPM2-AS (Ding et al., 2021), and LOC102724169 (Zhou et al., 2021). While these studies are a testimony to the potential of lncRNAs as modulators of cisplatin resistance, the work has not yet resulted in any clinically relevant therapies. Therefore, characterization of further lncRNAs is still needed, particularly in light of the many reported functions regulated by non-coding RNAs, including miRNAs and lncRNAs (Natarajan, 2016; DiStefano, 2017; Kwok et al., 2017; Balas and Johnson, 2018). For this study, we focused on the lncRNA HOTTIP because of its immense potential as a biomarker (Lian et al., 2016; Fan et al., 2018) but without any prior exploration in cisplatin resistance of ovarian cancer even though there is an indication for it role in cisplatin resistance of other human cancers (Yin et al., 2020).

For our study, we used a paired cell line model consisting of A2780 cells and their cisplatin resistant derivatives. These were commercially obtained and are thus excellent tools for such studies. A2780 cells are relatively sensitive to cisplatin and therefore this cell line model is appropriate for the studies focusing on cisplatin resistance of ovarian cancer cells. However, as with any study with cell lines, the results are always in question and therefore confirmation/validation in other similar cell lines is warranted. For this reason, we also used SK-OV-3 cells in our study. As compared to A2780 cells, SK-OV-3 cells are resistant to cisplatin and therefore the approach taken in this work, i.e., silencing of HOTTIP made sense in this cell line as well. Through the use of these two independent cell lines, we have presented novel data for a role of HOTTIP in cisplatin resistant ovarian cancer cells.

As a mechanism, we explored NF-κB pathway as well as the stem cells as they are regarded as attractive targets for anticancer therapy (Rizvi et al., 2021). The rationale was that there is evidence for stem cell inducing activity of HOTTIP in different other cancers (Fu et al., 2017; Luo et al., 2019). This makes sense because drug resistance is influenced by stem cell characteristics. Moreover, NF-κB pathway is intricately connected with stem cell phenotype and our results directly implicating HOTTIP in the activation of NF-κB as well as expression of stem cell marker genes raise the possibility of the involvement of these pathways as the mechanisms of action. While further elucidation of this mechanism was beyond the scope of our current work, this is an interesting lead for future investigations.

While the choice of lncRNA HOTTIP for its possible role in cisplatin resistance of ovarian cancer cells was based on a testable hypothesis, we used web-based predictive tools and also tested several other putative miRNA targets of lncRNA HOTTIP, in an attempt to pin-point miRNA(s) that the lncRNA HOTTIP might sponge with resulting effects on cisplatin resistance mechanism. For example, we screened miR-200 family miRNAs to which miR-205 belongs, and found a significant effect of miR-205 only. Other screened miRNAs included miR-615, miR-216a, miR-101, miR-148a, miR-150, etc. Of note, miR-615 has been one of the more consistent miRNA that HOTTIP has been shown to sponge. However, in our hands, miR-205 emerged as the more important target of HOTTIP. Further, we were able to show a direct effect of HOTTIP on the miR-205 target ZEB2 which further corroborates our findings and validates our choice of miR-205 as HOTTIP target. miR-205 also seems to play a role in proliferation and invasion of ovarian cancer cells as per the prior literature (Chu et al., 2018), in addition to the general interest in this miRNA in cancer (Ferrari and Gandellini, 2020).

Our results support an oncogenic activity of HOTTIP. We also establish sponging of miR-205 by HOTTIP. This reciprocal relationship means that miR-205 must be a tumor suppressor and indeed this is supported by available literature that this miRNA is a tumor suppressor in ovarian cancer (Qiao et al., 2020). Further, we also present evidence that miR-205 targets ZEB2. This would mean that ZEB2 should be oncogenic, which is also supported by reported literature (Li Q. et al., 2019). ZEB2 is also a marker of mesenchymal phenotype which again closely relates with stem cell properties thus further bringing the attention to stem cell phenotype as the underlying mechanism. Interestingly, we observed more robust effects of miR-205 manipulations on sensitivity to cisplatin as compared to HOTTIP silencing. This could be due to the inherent design of our experiments where we transfected cells with pre- or anti-miR-205 repeatedly (at least two or three time consecutively) as opposed to the siRNA against HOTTIP which was used just once. It is also possible that there might be a feedback or reciprocal relationship between HOTTIP and miR-205. These questions were not specifically answered in this study but would be interesting to elucidate. Regardless, we present compelling evidence for the role of HOTTIP-miR-205-ZEB2 axis in cisplatin resistance of ovarian cancer cells. This also underlines the enormous potential of lncRNAs such as HOTTIP as targets of therapy. The information should lead to future targeted therapies against cisplatin resistant ovarian cancers.

## Data Availability Statement

The original contributions presented in the study are included in the article/supplementary material, further inquiries can be directed to the corresponding author.

## Author Contributions

Y-JD led the study, arranged for resources, and drafted the manuscript. WF and YL conducted experiments, analyzed data, and edited the manuscript. All authors contributed to the article and approved the submitted version.

## Conflict of Interest

The authors declare that the research was conducted in the absence of any commercial or financial relationships that could be construed as a potential conflict of interest.

## References

[B1] Bai L. Wang A. Zhang Y. Xu X. Zhang X. (2018). Knockdown of MALAT1 enhances chemosensitivity of ovarian cancer cells to cisplatin through inhibiting the Notch1 signaling pathway. *Exp. Cell Res.* 366 161–171. 10.1016/j.yexcr.2018.03.014 29548748

[B2] Balas M. M. Johnson A. M. (2018). Exploring the mechanisms behind long noncoding RNAs and cancer. *Noncoding RNA Res.* 3 108–117. 10.1016/j.ncrna.2018.03.001 30175284 PMC6114262

[B3] Bergamini A. Pisano C. Di Napoli M. Arenare L. Della Pepa C. Tambaro R. (2017). Cisplatin can be safely administered to ovarian cancer patients with hypersensitivity to carboplatin. *Gynecol. Oncol.* 144 72–76. 28094039 10.1016/j.ygyno.2016.10.023

[B4] Chang H. Li B. Zhang X. Meng X. (2020). NCK1-AS1 promotes NCK1 expression to facilitate tumorigenesis and chemo-resistance in ovarian cancer. *Biochem. Biophys. Res. Commun.* 522 292–299. 10.1016/j.bbrc.2019.11.014 31761329

[B5] Chen Y. Li F. Li D. Liu W. Zhang L. (2021). Atezolizumab and blockade of LncRNA PVT1 attenuate cisplatin resistant ovarian cancer cells progression synergistically via JAK2/STAT3/PD-L1 pathway. *Clin. Immunol.* 227:108728. 10.1016/j.clim.2021.108728 33878452

[B6] Chi C. Mao M. Shen Z. Chen Y. Chen J. Hou W. (2018). HOXD-AS1 exerts oncogenic functions and promotes chemoresistance in cisplatin-resistant cervical cancer cells. *Hum. Gene. Ther.* 29 1438–1448. 10.1089/hum.2017.256 29896986 PMC12199627

[B7] Chu P. Liang A. Jiang A. Zong L. (2018). miR-205 regulates the proliferation and invasion of ovarian cancer cells via suppressing PTEN/SMAD4 expression. *Oncol. Lett.* 15 7571–7578. 29725462 10.3892/ol.2018.8313 PMC5920363

[B8] Dai C. Xu P. Liu S. Xu S. Xu J. Fu Z. (2021). Long noncoding RNA ZEB1-AS1 affects paclitaxel and cisplatin resistance by regulating MMP19 in epithelial ovarian cancer cells. *Arch. Gynecol. Obstet.* 303 1271–1281. 10.1007/s00404-020-05858-y 33151424

[B9] Ding Y. Tan X. Abasi A. Dai Y. Wu R. Zhang T. (2021). LncRNA TRPM2-AS promotes ovarian cancer progression and cisplatin resistance by sponging miR-138-5p to release SDC3 mRNA. *Aging (Albany NY)* 13 6832–6848. 10.18632/aging.202541 33621194 PMC7993682

[B10] DiStefano J. K. (2017). Long noncoding RNAs in the initiation, progression, and metastasis of hepatocellular carcinoma. *Noncoding RNA Res.* 2 129–136. 10.1016/j.ncrna.2017.11.001 30159431 PMC6084840

[B11] Fan Y. Yan T. Chai Y. Jiang Y. Zhu X. (2018). Long noncoding RNA HOTTIP as an independent prognostic marker in cancer. *Clin. Chim. Acta* 482 224–230. 10.1016/j.cca.2017.07.031 28778381

[B12] Ferrari E. Gandellini P. (2020). Unveiling the ups and downs of miR-205 in physiology and cancer: transcriptional and post-transcriptional mechanisms. *Cell Death Dis.* 11:980. 33191398 10.1038/s41419-020-03192-4 PMC7667162

[B13] Fu Z. Chen C. Zhou Q. Wang Y. Zhao Y. Zhao X. (2017). LncRNA HOTTIP modulates cancer stem cell properties in human pancreatic cancer by regulating HOXA9. *Cancer Lett.* 410 68–81. 10.1016/j.canlet.2017.09.019 28947139

[B14] Guo J. Pan H. (2019). Long noncoding RNA LINC01125 enhances cisplatin sensitivity of ovarian cancer via miR-1972. *Med. Sci. Monit.* 25 9844–9854. 10.12659/msm.916820 31865363 PMC6938651

[B15] He R. Zhu B. Liu J. Zhang N. Zhang W. H. Mao Y. (2021). Women’s cancers in China: a spatio-temporal epidemiology analysis. *BMC Womens Health* 21:116. 10.1186/s12905-021-01260-1 33743648 PMC7981806

[B16] Kwok G. T. Zhao J. T. Weiss J. Mugridge N. Brahmbhatt H. MacDiarmid J. A. (2017). Translational applications of microRNAs in cancer, and therapeutic implications. *Noncoding RNA Res.* 2 143–150. 30159433 10.1016/j.ncrna.2017.12.002 PMC6084838

[B17] Lee M. W. Ryu H. Song I. C. Yun H. J. Jo D. Y. Ko Y. B. (2020). Efficacy of cisplatin combined with topotecan in patients with advanced or recurrent ovarian cancer as second- or higher-line palliative chemotherapy. *Medicine (Baltimore)* 99:e19931. 10.1097/md.0000000000019931 32332673 PMC7440193

[B18] Li J. Yang S. Su N. Wang Y. Yu J. Qiu H. (2016). Overexpression of long non-coding. *Tumour Biol.* 37 2057–2065. 26341496 10.1007/s13277-015-3998-6

[B19] Li Q. Ma L. Wu Z. Wang G. Huang Q. Shen Z. (2019). Zinc finger Ebox binding homeobox 2 functions as an oncogene in human laryngeal squamous cell carcinoma. *Mol. Med. Rep.* 19 4545–4552. 30957184 10.3892/mmr.2019.10126 PMC6522803

[B20] Li Q. Zhang J. Zhou J. Yang B. Liu P. Cao L. (2018). lncRNAs are novel biomarkers for differentiating between cisplatin-resistant and cisplatin-sensitive ovarian cancer. *Oncol. Lett.* 15 8363–8370. 29805570 10.3892/ol.2018.8433 PMC5950027

[B21] Li Z. Niu H. Qin Q. Yang S. Wang Q. Yu C. (2019). lncRNA UCA1 mediates resistance to cisplatin by regulating the mir-143/FOSL2-signaling pathway in ovarian cancer. *Mol. Ther. Nucleic Acids* 17 92–101. 10.1016/j.omtn.2019.05.007 31234009 PMC6595407

[B22] Lian Y. Cai Z. Gong H. Xue S. Wu D. Wang K. (2016). HOTTIP: a critical oncogenic. *Mol. Biosyst.* 12 3247–3253. 10.1039/c6mb00475j 27546609

[B23] Liu E. Liu Z. Zhou Y. Mi R. Wang D. (2015). Overexpression of long non-coding RNA PVT1 in ovarian cancer cells promotes cisplatin resistance by regulating apoptotic pathways. *Int. J. Clin. Exp. Med.* 8 20565–20572. 26884974 PMC4723819

[B24] Liu R. Zeng Y. Zhou C. F. Wang Y. Li X. Liu Z. Q. (2017). Long noncoding RNA expression signature to predict platinum-based chemotherapeutic sensitivity of ovarian cancer patients. *Sci. Rep.* 7:18. 28154416 10.1038/s41598-017-00050-w PMC5428368

[B25] Luo H. Zhu G. Xu J. Lai Q. Yan B. Guo Y. (2019). HOTTIP lncrna promotes hematopoietic. *Cancer Cell* 36 645–659e8. 31786140 10.1016/j.ccell.2019.10.011 PMC6917035

[B26] Miao J. T. Gao J. H. Chen Y. Q. Chen H. Meng H. Y. Lou G. (2019). LncRNA ANRIL affects the sensitivity of ovarian cancer to cisplatin via regulation of let-7a/HMGA2 axis. *Biosci. Rep.* 39:BSR20182101. 31189742 10.1042/BSR20182101 PMC6609561

[B27] Natarajan V. (2016). Regulation of DNA repair by non-coding miRNAs. *Noncoding RNA Res.* 1 64–68. 10.1016/j.ncrna.2016.10.002 30159412 PMC6096415

[B28] Qiao B. Wang Q. Zhao Y. Wu J. (2020). miR-205-3p functions as a tumor suppressor in ovarian carcinoma. *Reprod. Sci.* 27 380–388. 10.1007/s43032-019-00047-y 32046433

[B29] Rizvi A. Farhan M. Nabi F. Khan R. H. Adil M. Ahmad A. (2021). Transcriptional control of the oxidative stress response and implications of using plant derived molecules for therapeutic interventions in cancer. *Curr. Med. Chem.* 10.2174/0929867328666210218110550 33602067

[B30] Sajadpoor Z. Amini-Farsani Z. Teimori H. Shamsara M. Sangtarash M. H. Ghasemi-Dehkordi P. (2018). Valproic acid promotes apoptosis and cisplatin sensitivity through downregulation of H19 noncoding RNA in ovarian A2780 cells. *Appl. Biochem. Biotechnol.* 185 1132–1144. 10.1007/s12010-017-2684-0 29468525

[B31] Shen F. Feng L. Zhou J. Zhang H. Xu Y. Jiang R. (2019). Overexpression of CASC11 in ovarian squamous cell carcinoma mediates the development of cancer cell resistance to chemotherapy. *Gene* 710 363–366. 10.1016/j.gene.2019.06.011 31181314

[B32] Taheri M. Shoorei H. Tondro Anamag F. Ghafouri-Fard S. Dinger M. E. (2021). LncRNAs and miRNAs participate in determination of sensitivity of cancer cells to cisplatin. *Exp. Mol. Pathol.* 8:104602. 10.1016/j.yexmp.2021.104602 33422487

[B33] Tan W. X. Sun G. Shangguan M. Y. Gui Z. Bao Y. Li Y. F. (2020). Novel role of lncRNA CHRF in cisplatin resistance of ovarian cancer is mediated by miR-10b induced EMT and STAT3 signaling. *Sci. Rep.* 10:14768. 32901049 10.1038/s41598-020-71153-0 PMC7478977

[B34] Torre L. A. Trabert B. DeSantis C. E. Miller K. D. Samimi G. Runowicz C. D. (2018). Ovarian cancer statistics, 2018. *CA Cancer J. Clin.* 68 284–296. 10.3322/caac.21456 29809280 PMC6621554

[B35] Vera O. Rodriguez-Antolin C. de Castro J. Karreth F. A. Sellers T. A. Ibanez I. (2018). de Caceres, An epigenomic approach to identifying differential overlapping and cis-acting lncRNAs in cisplatin-resistant cancer cells. *Epigenetics* 13 251–263. 10.1080/15592294.2018.1436364 29436261 PMC5997141

[B36] Wang D. Y. Li N. Cui Y. L. (2020). Long non-coding RNA CCAT1 sponges mir-454 to promote chemoresistance of ovarian cancer cells to cisplatin by regulation of surviving. *Cancer Res. Treat.* 52 798–814. 10.4143/crt.2019.498 32124583 PMC7373880

[B37] Wang F. Zhou J. Xie X. Hu J. Chen L. Hu Q. (2015). Involvement of SRPK1 in cisplatin resistance related to long non-coding RNA UCA1 in human ovarian cancer cells. *Neoplasma* 62 432–438. 10.4149/neo_2015_051 25967360

[B38] Wang H. Fang L. Jiang J. Kuang Y. Wang B. Shang X. (2018). The cisplatin-induced lncRNA PANDAR dictates the chemoresistance of ovarian cancer via regulating SFRS2-mediated p53 phosphorylation. *Cell Death Dis.* 9:1103. 30375398 10.1038/s41419-018-1148-y PMC6207559

[B39] Wang Y. Wang H. Song T. Zou Y. Jiang J. Fang L. (2015). HOTAIR is a potential. *Mol. Med. Rep.* 12 2211–2216. 10.3892/mmr.2015.3562 25824616

[B40] Wang Y. Wang X. Han L. Hu D. (2020). LncRNA MALAT1 regulates the progression and cisplatin resistance of ovarian cancer cells via modulating miR-1271-5p/E2F5 axis. *Cancer Manag. Res.* 12 9999–10010. 10.2147/cmar.s261979 33116856 PMC7567574

[B41] Wu Y. Zhou Y. He J. Sun H. Jin Z. (2019). Long non-coding RNA H19 mediates ovarian cancer cell cisplatin-resistance and migration during EMT. *Int. J. Clin. Exp. Pathol.* 12 2506–2515. 31934077 PMC6949588

[B42] Xu Q. F. Tang Y. X. Wang X. (2018). LncRNA EBIC promoted proliferation, metastasis and cisplatin resistance of ovarian cancer cells and predicted poor survival in ovarian cancer patients. *Eur. Rev. Med. Pharmacol. Sci.* 22 4440–4447. 30058681 10.26355/eurrev_201807_15495

[B43] Yan H. Xia J. Y. Feng F. Z. (2017). Long non-coding RNA ENST00000457645 reverses cisplatin resistance in CP70 ovarian cancer cells. *Genet. Mol. Res.* 23:16. 10.4238/gmr16019411 28128423

[B44] Yang L. Zhao H. Yin X. Liang H. Zheng Z. Shen Q. (2020). Exploring cisplatin resistance in ovarian cancer through integrated bioinformatics approach and overcoming chemoresistance with sanguinarine. *Am. J. Transl. Res.* 12 923–939. 32269724 PMC7137043

[B45] Yin F. Zhang Q. Dong Z. Hu J. Ma Z. (2020). LncRNA HOTTIP participates in cisplatin resistance of tumor cells by regulating mir-137 expression in pancreatic cancer. *Onco Targets Ther.* 13 2689–2699. 10.2147/ott.s234924 32280243 PMC7132030

[B46] Yu Y. Zhang X. Tian H. Zhang Z. Tian Y. (2018). Knockdown of long non-coding RNA HOTAIR increases cisplatin sensitivity in ovarian cancer by inhibiting cisplatin-induced autophagy. *J. BUON* 23 1396–1401. 30570864

[B47] Zhang C. Wang M. Shi C. Shi F. Pei C. (2018). Long non-coding RNA Linc00312 modulates the sensitivity of ovarian cancer to cisplatin via the Bcl-2/Caspase-3 signaling pathway. *Biosci. Trends* 12 309–316. 10.5582/bst.2018.01052 29952351

[B48] Zhang D. Ding L. Li Y. Ren J. Shi G. Wang Y. (2017). Midkine derived from cancer-associated fibroblasts promotes cisplatin-resistance via up-regulation of the expression of lncRNA ANRIL in tumour cells. *Sci. Rep.* 7:16231. 29176691 10.1038/s41598-017-13431-y PMC5701200

[B49] Zhang J. Liu J. Xu X. Li L. (2017). Curcumin suppresses cisplatin resistance development partly via modulating extracellular vesicle-mediated transfer of MEG3 and miR-214 in ovarian cancer. *Cancer Chemother. Pharmacol.* 79 479–487. 10.1007/s00280-017-3238-4 28175963

[B50] Zhang Y. Ai H. Fan X. Chen S. Wang Y. Liu L. (2020). Knockdown of long non-coding RNA HOTAIR reverses cisplatin resistance of ovarian cancer cells through inhibiting miR-138-5p-regulated EZH2 and SIRT1. *Biol. Res.* 53:18. 32349783 10.1186/s40659-020-00286-3 PMC7191713

[B51] Zheng Z. G. Xu H. Suo S. S. Xu X. L. Ni M. W. Gu L. H. (2016). The essential role of H19 contributing to cisplatin resistance by regulating glutathione metabolism in high-grade serous ovarian cancer. *Sci. Rep.* 6:26093. 27193186 10.1038/srep26093 PMC4872133

[B52] Zhou X. Liu M. Deng G. Chen L. Sun L. Zhang Y. (2021). lncRNA LOC102724169 plus cisplatin exhibit the synergistic anti-tumor effect in ovarian cancer with chronic stress. *Mol. Ther. Nucleic Acids* 24 294–309. 33850634 10.1016/j.omtn.2021.03.001 PMC8010577

[B53] Zhu M. Yang L. Wang X. (2020). NEAT1 knockdown suppresses the cisplatin resistance in ovarian cancer by regulating mir-770-5p/PARP1 axis. *Cancer Manag. Res.* 12 7277–7289. 10.2147/cmar.s257311 32884343 PMC7434570

[B54] Zou H. Li H. (2019). Knockdown of long non-coding RNA LINC00152 increases cisplatin sensitivity in ovarian cancer cells. *Exp. Ther. Med.* 18 4510–4516. 31777553 10.3892/etm.2019.8066 PMC6862479

[B55] Zou S. H. Du X. Sun F. D. Wang P. C. Li M. (2018). Cisplatin suppresses tumor proliferation by inhibiting autophagy in ovarian cancer via long non-coding RNA RP11-135L22.1. *Eur. Rev. Med. Pharmacol. Sci.* 22 928–935. 29509240 10.26355/eurrev_201802_14372

